# Acute low- compared to high-load resistance training to failure results in greater energy expenditure during exercise in healthy young men

**DOI:** 10.1371/journal.pone.0224801

**Published:** 2019-11-11

**Authors:** Diego T. Brunelli, Enrico A. R. Finardi, Ivan L. P. Bonfante, Arthur F. Gáspari, Amanda V. Sardeli, Thiago M. F. Souza, Mara P. T. Chacon-Mikahil, Claudia R. Cavaglieri

**Affiliations:** 1 Exercise Physiology Laboratory (FISEX)—Faculty of Physical Education, University of Campinas (UNICAMP), Campinas, São Paulo, Brazil; 2 Graduate Program in Gerontology–Faculty of Medical Sciences, University of Campinas (UNICAMP), Campinas, São Paulo, Brazil; Universidade Federal de Mato Grosso do Sul, BRAZIL

## Abstract

The objective of the present study was to verify the energy expenditure (EE), energy system contributions and autonomic control during and after an acute low-load or high-load resistance training (RT) protocol to momentary failure (MF) in young adults. Eleven young men (22 ± 3 yrs, 71.8 ± 7.7 kg; 1.75 ± 0.06 m) underwent a randomized crossover design of three knee extension acute protocols: a low-load RT [30% of their maximal strength (1RM); RT30] or a high-load RT (80% of 1RM; RT80) protocol, with all sets being performed to MF; or a control session (Control) without exercise. Participants were measured for EE, energy system contributions, and cardiac autonomic control before, during, and after each exercise session. Exercise EE was significantly higher for RT30 as compared to RT80. Furthermore, post measurements of blood lactate levels and the anaerobic lactic system contribution were significantly greater for RT30 as compared to RT80. In addition, parasympathetic restoration was lower for RT30 as compared to RT80. In conclusion, a low-load (30% 1RM) RT session produced higher EE during exercise than a high-load (80% 1RM) RT session to MF, and may be a good option for fitness professionals, exercise physiologists, and practitioners when choosing the optimal RT protocol that provides more EE, especially for those who want or need to lose weight.

## Introduction

Resistance training (RT) is known to promote several benefits for the practitioners such as increases in energy expenditure (EE), skeletal muscle mass, strength, and power and also reductions in fat mass, visceral and subcutaneous fat, inflammatory markers, lipid profile, and cardiometabolic risk factors [1]. Furthermore, it seems that RT performed with loads equal to or greater than 80% of 1 repetition maximal (1RM) increase the hypertrophic gains and muscle strength in a greater magnitude when compared to lower intensity protocols [2, 3].

On the other hand, studies have found that similar muscle hypertrophy and strength improvements can result from lifting loads to failure with higher (80% of 1RM) or lower (30% of 1RM) loads [4–7]. However, whether the magnitude of EE generated by low-load (30% 1RM) or high-load (80% 1RM) RT protocols is similar is still undetermined. Thus, it becomes clear that there is a need to investigate whether these RT protocols with different loads but similar muscle mass gains can provide additional EE during and after the training sessions.

In addition, the time to restore parasympathetic modulation after exercise indicates the time under exercise cardiac stress [8]. Autonomic nervous system (ANS) coordinates the cardiovascular adjustments required to supply the exercise metabolic demand and specific metabolic demands are identified by the ANS through different afferent stimuli, among which muscle metaboreflex plays an important role during muscle metabolites accumulation induced by RT protocols, mainly when it is performed to failure [9, 10]. Besides muscle metaboreceptors, many other neural mechanisms such as muscle pressor receptors, cardiopulmonary receptors, carotid and aortic chemo- and baroceptors conduct signals to cardiovascular control nuclei on the brain steam that after interactively processing these signals regulate the sympathetic and parasympathetic efferent neuronal activation or deactivation [11]. Thus, despite we might expect a linear association of EE and autonomic modulation, the different afferent mechanism the brain uses to identify the metabolic need in the body lead to different autonomic adjustments to exercise. In a previous study, Sardeli et al. [10] observed that a low-load RT protocol performed until failure promoted a delayed vagal restoration following an acute session when compared to a high-load RT protocol to failure; However, whether higher EE could be associated to the low- load RT protocol generating a delayed vagal restoration than the high-load RT protocol after the sessions is still unknown.

Since the knowledge of possible differences in EE, energy system contributions and cardiac autonomic control between a high-load or a low-load RT protocol to failure may help fitness professionals and/or exercise physiologists to choose the optimal RT protocol depending on the population considered, the purpose of this study was to compare the energy cost and cardiac autonomic recovery during and after two similar hypertrophic RT protocols [4–7] of low-load (30% 1RM) and high-load (80% 1RM), with all sets being performed until momentary failure (MF) [12]. In addition, since the amount of work performed within the set may contribute to the amount of EE [13] and to a delayed vagal restoration [10] following an RT session, we hypothesized that low-load RT protocol would produce greater EE during exercise and a delayed parasympathetic restoration than high-load RT protocol performed to momentary failure (MF).

## Materials and methods

### Participants

The disclosure of the project was made by folders and posters in the university campus and internet. Inclusion criteria were as follows: men with a non-active lifestyle (frequency of regular physical activity less than two sessions per week) who had not participated in regular resistance exercise programs for the previous 12 months according to the Baecke Habitual Physical Activity Questionnaire [14]. Exclusion criteria included the following: volunteers who presented in clinical evaluation (physical examination and resting ECG) any pathology or other complications that were risk factors in the practice of the proposed RT exercises.

Thirteen healthy young men (18–30 years old) with no experience in RT were recruited and assigned to a randomized, counterbalanced, crossover design of three acute protocols: a low-load (30% of their 1RM; RT30) or a high-load (80% 1RM; RT80) RT protocol, with all sets being performed to momentary failure (MF); or a control session without exercise (Control); however, two volunteers opted to discontinue their participation in the project for personal reasons, resulting in the final sample of 11 volunteers (Table 1). None of the volunteers were obese, diabetic or using any prescription drugs, supplements or others substances that may affect the present data. All volunteers in the present study were classified as sedentary or irregularly active [14].

**Table 1:** Participants’ baseline characteristics and dietary intake.

Age (years)	22 ± 3
Weight (kg)	71.8 ± 7.7
Height (m)	1.75 ± 0.06
Body mass index (kg/m^2^)	23.05 ± 2.35
Body fat (%)	16.9 ± 6.1
Fat free mass (kg)	59.5 ± 6.4
One-repetition maximum (kg)	93.1 ± 20.6
Total calories (kcal)	2041 ± 497
Proteins (g)	87.3 ± 31.5
Lipids (g)	81.8 ± 62
Carbohydrates (g)	238.7 ± 74.3

Mean ± SD (n = 11).

The experimental methods and procedures were all approved by the Research Ethics Committee of the State University of Campinas, Brazil.

All participants signed an informed consent document (written) approved by the local University Research Ethics Committee (Protocol nº 890.014).

### Experimental design

Prior to baseline testing all participants came to the laboratory and were submitted to two RT familiarization sessions, separated by 72h of rest between them, in order to be acquainted with the range of motion and the proper form for the leg extension machine RT exercise, familiarize themselves with the portable gas analyzer equipment (Oxycon, Carefusion Germany 234 GmbH, Hoechberg, Germany) while testing and all methodologies used in the present study. After 72h of the last familiarization session, volunteers performed the test and re-test of 1RM on the leg extension machine, with a 72h interval between them. One week after the re-testing of 1RM, volunteers underwent the RT30 or RT80 protocol, with all sets being performed to MF; or a control session without exercise (Control), according to the randomization performed.

The acute RT protocols were composed of performing three sets of leg extension machine using the intensity corresponding to the session (30% or 80% of 1RM), with all sets being performed until MF [12] and with one and a half minutes of rest applied between each set. In the Control, volunteers performed all the procedures for determination of EE; however, they remained seated quietly in the leg extension machine during the time of exercise (approximately 8 to 10 min). After the end of the acute sessions, volunteers remained lying on an examination couch in the room for 60 minutes and expired air was collected continuously.

Before the acute sessions, resting EE (REE) was assessed for 30 minutes with the volunteers lying on an examination couch and resting. In addition, volunteers were requested to record all the foods and beverages ingested in the day before the first acute session and instructed to match the same dietary intake patterns before the subsequent acute sessions. During all sessions, breath-by-breath gas exchange was collected with a portable gas analyzer and blood lactate samples were collected to determine REE, energy system contributions, exercise EE (Exercise EE), excess post-exercise oxygen consumption (EPOC), and total EE of the session (Total EE). Blood lactate samples were collected before (PRE) and after 3 (3min), 5 (5min), 7 (7min), and 60 (60min) minutes of the acute protocols. Heart rate variability (HRV) was recorded before (PRE), post 10 minutes (10min), and post 45 minutes (45min). In addition, subjective perception of effort [15] was applied in the end of the session.

For all EE quantifications, measurements were taken between 7:00–12:00 a.m. in a controlled temperature and humidity environment where the noise was minimal. In order to obtain the closest measurement of their physiological conditions, participants were instructed to sleep well prior to the sessions and to refrain from consuming alcohol and caffeine in the 24 hours preceding the measurements and any physical activity for the 72 hours prior to measurements. In addition, all participants fasted for at least 7 hours before the Control, RT30, or RT80 acute sessions thereby avoiding any variation in EE from feeding; however, water intake was encouraged; thus it is believed that all participants entered the laboratory in a hydrated state. Furthermore, a period of seven days of rest without exercise was used between the experimental protocols to wash out the effects of muscle recuperation.

### Anthropometric measures and body composition

Height was measured using a wall-mounted stadiometer with a precision of 0.1 cm, and weight was taken using a calibrated manual scale (Filizola® S.A., São Paulo, SP, Brazil) with a precision of 0.1 kg. The body composition of the volunteers was estimated by plethysmography in the Bod Pod ^™^ (COSMED USA, Inc., Concord, CA) body composition system. The same investigator performed all measurement assessments.

### Dietary intake

Food records were given to the particpants by trained researches who instructed them individually through a presentation of an already completed model food record and photographs of model home measures. Food records for total caloric intake and amount of macronutrients (carbohydrates, lipids, and proteins) were analyzed using the DietPro software program (version 5i).

### Blood lactate samples and analyses

For analysis of blood lactate levels, samples (25 μL) of peripheral blood from the distal phalanges of the hand were collected using lancets (Accu-Chek Safe-T-Pro Uno, Roche Diagnostics GmbH, Indianapolis, IN, USA) and microcapillary tubes. All blood samples were placed in microtubes containing a similar volume (25 μL) of a 1% NaF solution. Plasma was separated by centrifugation of the samples for 10 minutes at 5,000rpm and stored at -80°C for subsequent analysis. Blood lactate levels were determined using a spectrophotometer (ELx800, Biotek, Winooski, USA) and commercially available kits (Biotecnica, Varginha, Brazil). The peak lactate level was determined by the highest lactate level value found in the three measurements (3min, 5min and 7min) assessed after the acute RT protocols or Control.

### Maximal strength assessments

Maximal strength was measured by a one-repetition maximum (1RM) test performed on leg extension machine (Johnson SL153 leg extension machine, Johnson Health Tech. Co., Ltd.), according to descriptions by Brown and Weir [16]. All participants were tested, at baseline, in two separated sessions (test-retest) with 72-h rest between them. To determine the results of the1RM tests at baseline, we used the value of the highest load obtained after the test-retest. The coefficient of variation and the intraclass correlation coefficient of the 1RM test-retest for leg extension machine were 5.33% and 0.93, respectively.

### Acute resistance training protocols

Acute RT protocols comprised performing three sets of knee extension machine (Johnson SL153 leg extension machine, Johnson Health Tech. Co., Ltd.) according to the intensity of the session: low-load (30% of their 1RM; RT30) or high-load (80% of their 1RM; RT80), with all sets being performed to MF and with one and a half minutes of rest applied between each set. The participant started extending the knee from the flexed knee position (~90° knee joint angle; concentric phase) until full extension (~0° knee joint angle), and then flexed the knee (eccentric phase) returning to the ~90° knee joint angle in the knee extension machine. The failure was recognized when the range of motion adopted in the present study to perform the exercise (at least 81 degrees during the concentric and eccentric phases) was not completed, where the range of motion was identified from a hand goniometer to check the angle of extension of the knee, and a metric tape positioned on the side of the equipment to check the position of the weight when the knee was extended [12]. The execution speed of the exercises was one second in concentric action and one second in eccentric action, controlled by a metronome, the exercise was not interrupted by the decrease of the execution speed.

The number of repetitions of each set was recorded and the volume of each set was calculated by multiplying the number of repetitions by the load. Afterwards, total volume was calculated as the sum of each set´s volume. All the acute RT protocols were based on the descriptions by Burd et al. [4], Mitchell et al. [5], Morton et al. [6], and Jenkins et al. [7]; thus it is believed that the acute RT protocols performed in the present study can promote similar muscle hypertrophy and strength gains if performed for chronic periods.

### Energy expenditure data collection and calculation

REE was calculated by the area under the oxygen uptake (VO_2_) curve during the central 20 minutes of the 30 minutes collected from resting, where the initial 5 minutes and 5 final minutes were excluded to avoid fluctuations. The aerobic energy system was calculated by the VO_2_ area over time during exercise from which VO_2_ from resting was subtracted. To estimate anaerobic alactic energy system we used an exponential model to fit the initial 7 minutes from VO_2_ recovery period, considered the post-exercise fast VO_2_ kinetics, acc. To calculate anaerobic lactic energy system the lactate accumulation (peak lactate minus resting lactate) was multiplied by the oxygen equivalent (3 ml O_2_.kg-1) and by the participant’s body mass. Exercise EE was calculated as the sum of the three energy systems. EPOC was calculated by the area under the 53 minutes remaining of the VO_2_ recovery period curve, i.e., 60 minutes of recovering minus the first 7 minutes utilized in the anaerobic alactic calculation. The Total EE was calculated by the sum of the Exercise EE and EPOC. All the variables for energy system contributions were estimated according to Bertuzzi et al. [17]. In addition, the area under the VO_2_ curve calculations (trapezoidal method) and energy system contributions estimation were performed using GEDAE-LaB software tools (http://www.gedaelab.org/) and Total EE was calculated using Excel software (Microsoft Corporation, California, USA)

### Heart rate variability

Continuous inter-beat (RR) intervals were acquired before and after one hour recovery in supine position using a Polar S810i heart rate monitor (Polar Electro, Kempele, Finland) and Polar ProTrainer 5 software (version 4.0. Kempele, Finland) [18] and analyzed following linear interpolation of adjacent beats in Kubios HRV software (Version 2.1, Biosignal Analysis and Medical Imaging Group, Kuopio, Finland) [19]. The time and frequency domains from linear and the non-linear indexes of HRV were analyzed. Among time domain indices, mean RR interval (RRi), standard deviation of all normal RR intervals (SDNN), and square root of the mean squared differences of successive RR intervals (RMSSD) were analyzed as representatives of parasympathetic modulation [20, 21]. Frequency domain indices were derived by a fast Fourier transform, which included low frequency (LF: 0.04–0.15 Hz) and high frequency (HF:0.15–0.4 Hz). HF represents parasympathetic modulation, as seen it is almost entirely mediated by the vagus nerve [21]. We opted to use LF in normalized units (LFnu), considering the normalization process tend to minimize the effect of variations in total power on its value; however, LFnu is influenced by parasympathetic and sympathetic modulation [20, 21]. Total power (TP) of the frequencies was used as a global marker of parasympathetic modulation [20–22].

### Statistical analysis

The sample size required was estimated using G*Power software (version 3.1.9.2), with data from the previous study comparing energy expenditure of low- vs. high-load single set resistance exercise [13]. A priori power analysis using an alpha level of 0.05 and an expected power of 0.8 suggested a sample size of 11 participants to achieve a statistical significant difference between low-load vs. high-load in this variable. Data distribution was tested by the Shapiro-Wilk test. The Student paired T-test was used to verify differences between RT30 and RT80 exercise total volume and Borg´s subjective perception of effort score. The one way ANOVA for repeated measures, followed by Tukey post hoc test, were performed to verify differences between conditions (RT30 vs. RT80 vs. Control) for energy system contributions, Exercise EE, EPOC, and Total EE. To identify differences between moments and conditions for blood lactate levels and log transformed heart rate variability variables, we used a two-way ANOVA for repeated measures. When significant moments X conditions interactions were detected, the Tukey post hoc test was applied to determine the source of significance. The level of significance was set at p ≤ 0.05 for all statistical comparisons. The software used for all analyses was Statistica 6.0 (StatSoft.inc, Tulsa, USA). All data are presented in terms of values of mean ± SD.

## Results

Total repetitions for each set was significantly higher in all sets for RT30 protocol (Set 1: 36 ± 9; Set 2: 26 ± 6; Set 3: 21 ± 6 repetitions) than the RT80 protocol (Set 1: 9 ± 3; Set 2: 8 ± 2; Set 3: 7 ± 2 repetitions) (p = 0.0001 to all comparisons). In addition, total volume was significantly higher in the RT30 protocol (2301.4 ± 631.1 kg) than the RT80 protocol (1828.1 ± 690.4 kg) (p = 0.0571). However, no significant difference was found for Borg's subjective perception of effort between RT30 (17 ± 2) and RT80 (16 ± 2) after the end of the acute RT protocols (p > 0.05). In addition, no difference was found for REE before all sessions (Control: 25.9 ± 4.5 Kcal; RT30: 24.7 ± 4.5 Kcal; RT80: 26.5 ± 4.6 Kcal; p > 0.05).

**Fig 1:**
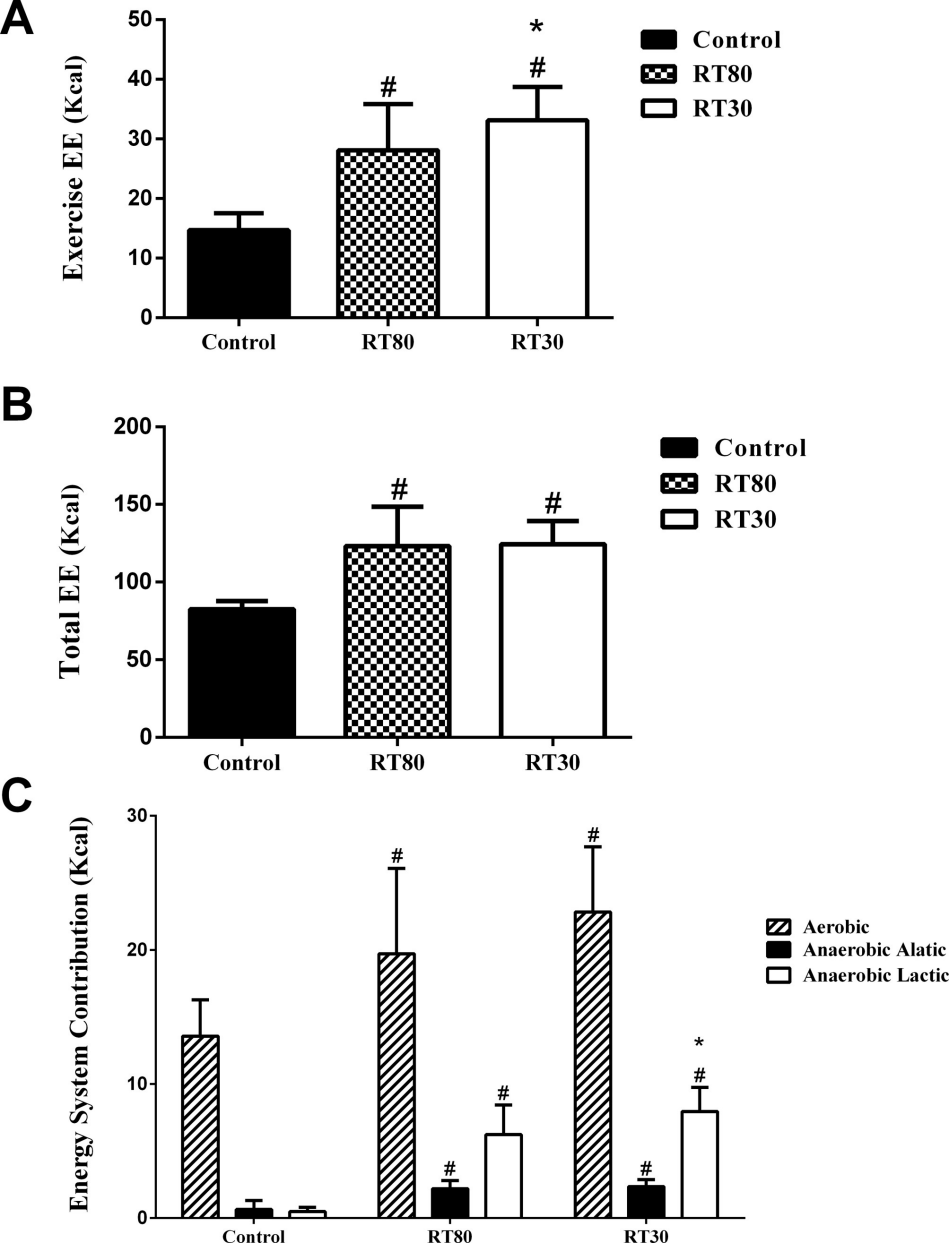
**Energy expenditure during exercise (Exercise EE; A), Total energy expenditure (Total EE; B) and Energy system contribution (C) from a low-load (30% of 1RM; RT30) or a high-load (80% of 1RM; RT80) RT protocol performed to momentary failure or a control session without exercise (Control).** #significantly different from Control. *significantly different from RT80. Mean ± SD (n = 11; *p ≤ 0*.*05*). Individual data points presented in S1 Dataset.

Exercise EE, Total EE and energy system contributions are presented in Fig 1. As expected, Exercise EE for both RT30 (*p = 0*.*0001*) and RT80 (*p = 0*.*0001*) were greater as compared to Control (Fig 1A). Furthermore, Exercise EE was significantly higher for RT30 as compared to RT80 (*p = 0*.*0243*; Fig 1A). Total EE was significantly higher for RT30 (*p = 0*.*0001*) and RT80 (*p = 0*.*0001*) as compared to Control (Fig 1B), although no significant difference was found for Total EE between RT30 and RT80 (*p = 0*.*9724*; Fig 1B). With respect to the energy system contributions, as expected, the aerobic, anaerobic alactic and anaerobic lactic systems contribution were significantly higher for RT30 (*p = 0*.*0001*; *p = 0*.*0001*, and *p = 0*.*0001*, respectively) and RT80 (*p = 0*.*0022*; *p = 0*.*0001*, and *p = 0*.*0001*, respectively) when compared to Control (Fig 1C). Also, the anaerobic lactic system contribution was higher in the RT30 protocol than in the RT80 protocol (*p = 0*.*0476*; Fig 1C). There were no significant differences for aerobic (*p = 0*.*1349*) and anaerobic alactic system (*p = 0*.*7936*) between RT30 and RT80 (Fig 1C).

**Fig 2:**
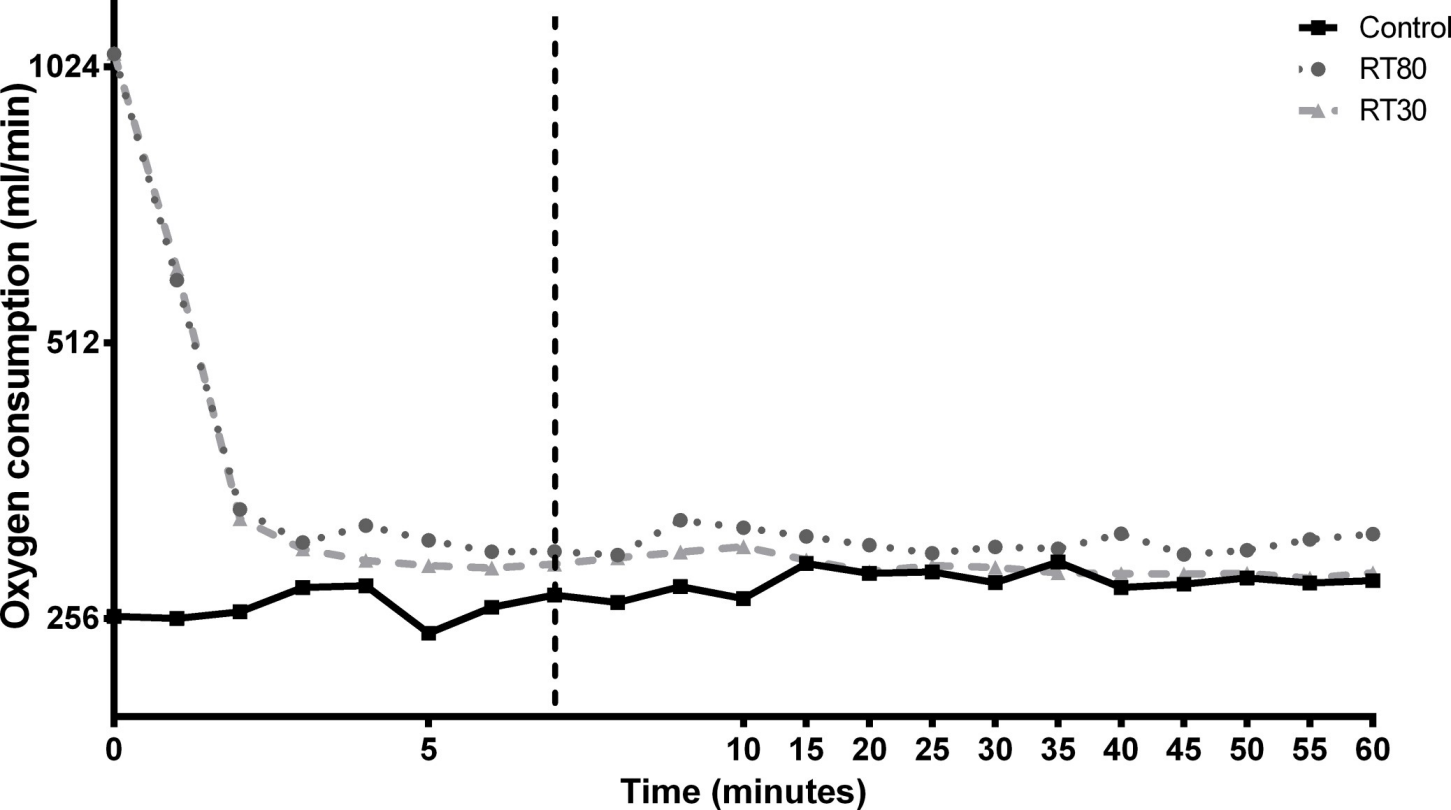
Schematic evaluation of excess post-exercise oxygen consumption (EPOC) from a low-load (30% of 1RM; RT30) or a high-load (80% of 1RM; RT80) RT protocol performed to momentary failure or a control session without exercise (Control). The dashed line represents the initial 7 minutes of recovery used for the calculation of the anaerobic alactic system contribution of the exercise. Individual data points presented in S1 Dataset.

EPOC for RT80 (95.1 ± 18.4 Kcal) was greater as compared to control (75.8 ± 7.6 Kcal *p = 0*.*0260*). However, no significant difference was found for EPOC between RT30 (91.4 ± 10.6 Kcal) vs. Control (*p = 0*.*1212*) nor for RT30 vs. RT80 (*p = 0*.*7238*). Fig 2 represents the schematic evaluation of EPOC after the acute protocols.

**Fig 3:**
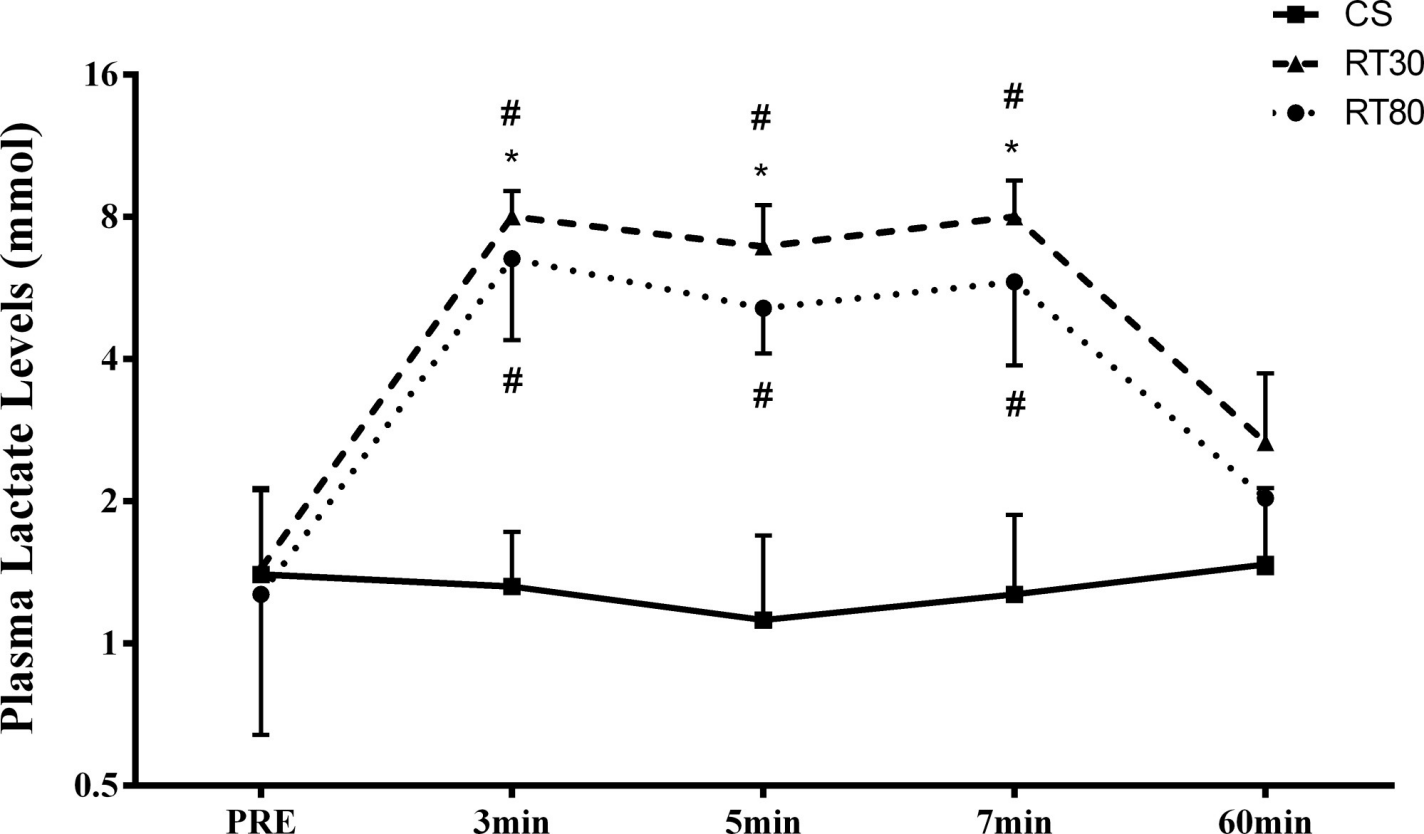
Blood lactate levels before (PRE) and after 3 (3min), 5 (5min) and 7 (7min) minutes after a low-load (30% of 1RM; RT30) or a high-load (80% of 1RM; RT80) RT protocol performed to momentary failure or a control session without exercise (Control). #significantly different from Control. *significantly different from RT80. Mean ± SD (n = 11; *p ≤ 0*.*05*). Individual data points presented in S1 Dataset.

Fig 3 represents the blood lactate levels before and after the acute protocols. Significant increases in lactate levels were found in the 3min, 5min and 7min post the exercise period for RT30 and RT80 as compared to Control (*p < 0*.*001* for all comparisons). Furthermore, increased lactate levels were significantly higher for RT30 in the 3min (*p = 0*.*0343*), 5min (*p = 0*.*0030*) and 7min (*p = 0*.*0002*) post exercise than RT80 (Fig 3).

Table 2 shows the HRV before and after the experimental sessions. There was lower parasympathetic modulation (SDNN and RMSSD) in 10min compared to PRE and both RT protocols different of Control at 10min (Table 2). For these parasympathetic indexes, at 45min, RT80 was not different from PRE, while RT30 was still different from PRE and Control for RRi and tended to be different from PRE for RMSSD (*p = 0*.*08*). In addition, the reduction of total power in 10min was considerable for RT30 compared to RT80.

**Table 2:** Heart rate variability before (PRE) and after 10 (10min) and 45 (45min) minutes after a low-load (30% of 1RM; RT30) or a high-load (80% of 1RM; RT80) RT protocol performed to momentary failure or a control session without exercise (Control).

	RRi (ms)	SDNN	RMSSD	HF (ms^2^)	TP	LF nu	HF nu
**Control**							
PRE	1048 ± 165	87 ± 32	94 ± 54	3635 ± 2924	7157 ± 4753	37 ± 20	63 ± 20
10min	1087 ± 165	96 ± 31	113 ± 54	4569 ± 3621	8226 ± 4756	36 ± 20	64 ± 20
45min	1088 ± 166	99 ± 43	107 ± 61	4061 ± 4033	9084 ± 6703	42 ± 17	58 ± 17
**RT80**							
PRE	1094 ± 174^a^	93 ± 31^a^	106 ± 45^a^	3757 ± 2596^a^	9500 ± 6619^a^	42 ± 24	58 ± 24
10min	907 ± 117^#^	56 ± 15^#^	46 ± 26^#^	1006 ± 1196^#^	3314 ± 1940^#^	56 ± 24	44 ± 24
45min	1015 ± 159^a^	77 ± 29^a^	79 ± 42^a^	2495 ± 2176^a^	5866 ± 3249^a^	47 ± 20	53 ± 20
**RT30**							
PRE	1074 ± 188^a^	98 ± 44^a^	115 ± 76^a^	5746 ± 6747^a^	9664 ± 7931^a^	41 ± 25	59 ± 25
10min	838 ± 152^#^	49 ± 31^#^	45 ± 40^#^	1042 ± 1774^#^	2670 ± 4393^#^^,^*	47 ± 12	53 ± 12
45min	948 ± 154^a^^,^^b^^,^^#^	82 ± 45^a^	80 ± 66^a^	3072 ± 4512^a^	7667 ± 9713^a^	47 ± 18	53 ± 1

RRi: mean RR interval; SDNN: standard deviation of all normal RR intervals; RMSSD: square root of the mean squared differences of successive RR intervals; HF: high frequency; TP: total power; LFnu: low frequency in normalized units; HFnu: high frequency in normalizes units.

^a^ Significantly different from 10min

^b^ Significantly different from PRE

^#^ Significantly different from Control

* Significantly different from RT80. Mean ± SD (n = 11; *p ≤ 0*.*05*).

## Discussion

Studies have demonstrated that RT increases EE both during [23, 24] and immediately post the exercise protocol [25]. To determine whether a low-load (30% of 1RM, RT30) or a high-load (80% of 1RM; RT80) RT protocol, with all sets being performed until MF, can provide different EE during and after an acute session, we tested the energy cost and the energy system contributions in young, healthy and sedentary men. In accordance with our initial hypothesis, the RT30 protocol produced greater EE during exercise as compared to RT80; however, EPOC did not differ between the RT protocols to MF. In addition, we show here that although both protocols produce similar total EE, the RT30 may induce a delayed vagal restoration after the end of the acute sessions.

It has been reported that EE increases as the intensity of RT increases, especially when total volume is matched [26]. However, in a well-controlled study, Mazzetti et al. [23] found no significant differences in total EE after four RT protocols: a light (48% of 1RM), moderate (60% of 1RM), heavy (70% of 1RM) or a heavy with loads equalized to moderate and light RT protocols, concluding that exercise intensity in RT did not affect total EE. In the present study, we observed no difference in total EE between RT30 and RT80, regardless of the fact that RT30 had a total volume of the session significantly higher than the RT80. Taking this into consideration, our results also suggest that RT intensity (load per repetition) may not influence total EE when sets are performed until MF, independent of the equalization in the total volume of the session.

As observed in previous studies, when a single RT set is performed until failure, a lower weight lifted should result in a greater number of repetitions and a heavier weight should result in fewer repetitions [6, 7, 13]. In addition, Scott et al. [13] observed that the energy cost of a single set of bench press performed until the volitional fatigue was higher when loads are performed with lower intensities (37%, 46% or 56% of 1RM) as compared with heavy intensities (70%, 80% or 90% of 1RM), concluding that the amount of work performed within the set may have contributed to the amount of EE during the experimental period; however, this was not sufficient to promote significant alterations in the EPOC data. In the present study, using three sets instead of one and a control session without exercise, both RT30 and RT80 with sets being performed until the MF were able to increase EE during exercise. We also observed that the amount of work performed during RT30 may have promoted a higher contribution from the anaerobic lactic system, generating a greater metabolic perturbation evidenced by greater lactate levels and resulting in a increased exercise EE when compared to RT80 protocol; however, the total amount of EE did not differ between RT30 and RT80, since only RT80 had significantly different EPOC from the Control demonstrating a compensatory effect.

Taking this into account, our results suggest that, when performed until failure, lactate accumulation and clearance is higher during a low-load RT protocol, and this could reflect the increased contribution of the anaerobic lactic system for increasing exercise EE as compared to a high-load RT protocol. Thus, the use of a multiple-sets RT program with low-load and with sets being performed to fatigue seems to be more beneficial to promoting higher rates of EE for those who want or need to lose weight.

Following this higher anaerobic lactic contribution for the higher EE during RT30, this protocol stimulated a lower parasympathetic restoration compared to RT80. We suggest that the higher volume of RT30 contributes to a higher metabolite accumulation such as lactate, which in turn stimulates muscle metaboreceptors and other chemoreceptors leading to its lower parasympathetic modulation [11]. Although higher load exercise could lead to higher sympathetic modulation, when the same RT volume is maintained [27], RT protocols to failure lead to higher sympathetic modulation and slower parasympathetic restoration during recovery [10]. Thus, comparing high-load and low-load RT protocols until failure, the metabolic accumulation (as measured by the blood lactate levels) from higher volume (during RT30) may contribute to parasympathetic recovery.

In contrast with what was expected, the RT protocol that prompted higher EE during exercise and worse parasympathetic restoration (RT30) did not lead to higher increases in EPOC [28]. Considering the delayed parasympathetic recovery for RT30, we speculate that this protocol may have been more efficient regarding exercise increases in sympathetic modulation, blood supply (we did not measured these factors in the present study) and energy production during exercise, which preserved the energetic storage and reduce the demand for EPOC [29]. In fact, higher sympathetic outflow during exercise enables higher oxygen consumption [28, 30], which likely occurs in low-load RT protocols [13], such as the RT30 used in the present study.

In addition, we also suggest that a low-load RT protocol until failure is physiologically more efficient to cardiovascular function because the higher dynamic component may facilitates the local vasodilation (functional sympatholysis), the venous return and mobilizes blood from the splanchnic area to exercised muscles to a higher extent [10]. Furthermore, previous studies that have observed an increased EPOC with higher EE and glycolytic demand probably found these results due to the inclusion of post-exercise fast VO_2_ kinetics in the EPOC calculation [26] which is the most O2 costly phase and closely represents the anaerobic contribution of exercise [17, 31–34].

In our study we analyzed both fast and slow components of EPOC, whereas the fast component was analyzed in the first 7 minutes post exercise and considered the anaerobic alactic energy system contribution of the exercise. The remaining 53 minutes were considered as the slow EPOC component. The fast component can be considered as a good measurement of the anaerobic alactic contribution, being responsible for the restoration of muscle adenosine triphosphate (ATP) and creatine phosphate stores [26, 30]. While the slow component is not yet well understood, it can be considered as a replenishment of oxygen stores in blood and muscle, lactate removal, and increased body temperature, circulation and ventilation. An increased triglyceride/fatty acid cycling, and a shift from carbohydrate to fat as substrate source, may explain a substantial part of the prolonged EPOC component after exhaustive exercise [26, 29]. As observed in our results, higher intensity training has a better effect for increasing EPOC versus lower intensity training, even with differences in the total volume of the session. This supports previous studies showing that a more intense exercise has better effects on EPOC when volumes are matched (Thornton & Potteiger, 2002); however, little is known when exercises are not matched [29]. Nevertheless, it seems that EPOC after RT is influenced by the intensity of the training and not by the total volume of training [35].

It is important to acknowledge that the number of sets and RT intensities used in the present study is based on previous studies that demonstrated similar muscle hypertrophy and strength improvements when lifting loads to failure with higher (80% of 1RM) or lower (30% of 1RM) loads [4–7]. In addition, EE rates from our data were from an acute perspective and using only one RT exercise. Future studies comparing the energy cost of a single RT session with low or high loads until failure could be conducted using more exercises or applying the same procedures as used in the present study to experienced RT individuals, overweight/obese or older participants. To this end, we recognize limitations of energy system contributions and energy expenditure estimations on intermittent exercises; however, the calculation approach used in this study can be considered a good option for calculating the EE of the organism as a whole, at least until the emergence of a gold standard [17].

In conclusion, a low-load (30% of 1RM) RT session produced higher EE during exercise as compared to a high-load (80% of 1RM) RT session with exercises being performed to the point of MF in young, healthy and sedentary men. These results can aid fitness professionals and/or exercise physiologists when choosing the optimal RT protocol that provides more EE without the expectation that strength or muscle mass gains would be compromised, especially for those who want or need to lose weight. However, the greater glycolytic contribution of a low-load RT session resulted in a delayed parasympathetic return; Thus, the magnitude of cardiovascular challenge should also be considered.

## Supporting information

S1 Dataset (XLSX)
